# Do community medicine residency trainees learn through journal club? An experience from a developing country

**DOI:** 10.1186/1472-6920-6-43

**Published:** 2006-08-22

**Authors:** Saima Akhund, Muhammad Masood Kadir

**Affiliations:** 1 Human Development Programme, Aga Khan University, Karachi, Pakistan; 2 Department of Community Health Sciences, Aga Khan University, Karachi, Pakistan

## Abstract

**Background:**

Journal clubs are an internationally recognized teaching tool in many postgraduate medical education fields. In developing countries lack of funds for current print materials may have limited journal club use. But with advancing information technology trainees in developing countries increasingly have more access to high quality journals online. However, we are aware of no studies describing journal club existence and effectiveness in postgraduate medical training in Pakistan. Also we have found no published effectiveness studies of this teaching modality in Community Medicine (Public Health) in any country. This study evaluated the effectiveness of Community Medicine (Public Health) Resident Journal Club (CMR-JC) in Aga Khan University, Pakistan using international criteria for successful journal clubs (2 years continuous existence and more than 50% attendance) and examining resident and alumni satisfaction.

**Methods:**

Journal club effectiveness criteria were searched using electronic search databases. Departmental records were reviewed from September1999–September 2005. Ninety percent of residents and alumni of Community Medicine Residency Programme participated voluntarily in a confidential survey.

**Results:**

The CMR-JC was regularly conducted. More than 95% of residents attended. (Total residents in the CMR-Programme: 32). Twenty-seven out of 29 current residents/alumni responded to the anonymous questionnaire. Acquisition of critical appraisal skills (23 respondents) and keeping up with current literature (18 respondents) were the two most important objectives achieved. Respondents recommended improved faculty participation and incorporating a structured checklist for article review.

**Conclusion:**

CMR-JC fulfils criteria for effective journal clubs. Residents and alumni agree CMR-JC meets its objectives. Incorporating suggested recommendations will further improve standards. The journal club learning modality should be included in residency training programs in developing countries. Effective use of online resources to support journal clubs is demonstrated as a successful alternative to excessive expenditure for obtaining print journals. Those trying to start or improve journal clubs can benefit from our experience.

## Background

A journal club can be defined as a group of individuals who meet regularly to discuss articles in current medical literature [1]. The earliest reference to a journal club is found in Sir James Paget's memoirs and letters (1835–1854) in which he described how a small group of students near St. Bartholomew's Hospital London met in a room over a baker's shop to read journals. Evidence of the first formal journal club comes from McGill University Montréal where, in 1875, William Osler found a way to make expensive periodicals affordable by organizing with fellow students to purchase expensive journals at a group rate [2].

At the start journal clubs helped students stay current with medical literature. Later, they improved acquisition of knowledge in clinical epidemiology, biostatistics, research design, and more recently in teaching critical appraisal skills [3-8]. Studies show that journal clubs promote critical thinking, reading habits and strengthening of collegial relationships. [3,6,9-11]. The journal club also has been advocated as a bridge between research and practice, hence facilitating better practice of evidence based medicine [12-17]. Current internet technology has added another dimension to traditional journal club, where online discussions are the main stay of journal club [18,19].

Many clinical disciplines report using journal clubs to train postgraduate trainees in relevant specialties. At present journal clubs are found in Medicine (and allied fields of Internal Medicine, Palliative Care, Family Medicine, Emergency Medicine and Critical Care, Cardiology, Paediatrics, Ophthalmology, Physical Medicine and Rehabilitation), Surgery (and allied fields of Obstetrics and Gyneacology, Orthopeadics, Neurosurgery, Hand surgery), Psychiatry, Geriatrics, Nursing, and Health Care Management [16,20-29]. Benefits of a journal club exercise are even documented at medical undergraduate level [30].

While a 1999 article from the Royal College of Physicians London illustrates the popularity of journal clubs in public health medicine related to clinical practice, [31] the existence of journal clubs in the discipline of Community Medicine (Public Health) has been documented sparsely. In Pakistan, the effectiveness of journal clubs has so far not been evaluated in any postgraduate medical education program.

In order to find criteria of effective journal clubs, articles were identified through electronic searches using PubMed, and Cochrane retrieval systems. Search terms used were "journal club", "effectiveness", "public health" and "post graduate medical education". Reference lists of known systematic reviews were also searched in this connection besides hand search of library periodicals [1,32-36].

The following criteria and associated factors of effective journal clubs were found:

### Criteria for effective journal club

- More than 2 years of existence without periodic abandonment

- More than 50 percent attendance of the expected audience

### Factors associated with effective journal club (listed in random order)

- Explicit written learning objectives

- Having a designated club leader

- Mandatory attendance

- Formal teaching of critical appraisal skills

- Journal club independent of faculty journal club

- Regular attendance by faculty

- High value given by program director

- Smaller residency programme (12 or less residents)

- Incorporation of adult learning principles

- Provision of free food

- Use of a structured checklist for article review

### Study objectives

The purpose of this study was to determine the effectiveness of the CMR-JC currently conducted in a Pakistan medical university by:

1) Comparing the criteria of effective journal clubs in the international literature with that of the CMR-JC

2) Seeking evidence of resident and alumni satisfaction with CMR-JC

### Community Medicine resident journal club (CMR-JC)

#### Target audience

CMR-JC is offered by the Community Medicine Residency Program (Community Health Sciences Department) of a Pakistan medical university. The target audience is Community Medicine Residents (equivalent to post graduate trainees in Public Health). These trainees already have a MBBS degree.

#### Goals

The goals of CMR-JC are to:

• increase awareness of residents regarding important national and international public health issues

• teach residents critical appraisal skills

• meet core competencies required by the College of Physicians and Surgeons Pakistan, the program accreditation body

• assess residents' knowledge and expertise over time in raising public health issues, utilizing public health tools and developing strategies to address issues.

#### Format and environment

The CMR-JC began in September 1999. It is conducted once every week for one hour. Attendance is mandatory for all Community Medicine Residents but students and faculty from other departments may also attend. On average there are 12 residents in the program, thus each resident is required to present every twelfth week. A presentation is followed by an interactive discussion. After the session, the presenting resident hosts a breakfast for participants.

#### Coordination

Senior residents of the program act as journal club coordinator on a rotational basis. Their responsibilities include helping other residents in article selection, preparing presentations, organizing mock presentations before the actual presentation day, ensuring the selected article reaches every one in the department at least two days prior to the meeting, and maintaining a record of journal club presentations. The journal club coordinator (resident) is guided by the coordinator and director of residency program.

#### Selection of presentation topic

The residents choose original articles of public health importance published within the past three years in indexed journals. Residents can also present their research work and experiences of placement done or training attended. Sometimes alumni of the program are invited to share important research they have conducted.

#### Structure of presentation

The resident first presents the rationale for choosing a particular topic by summarizing a background search indicating the global and local burden of the problem. Then the article is discussed according to Introduction, Methodology, Results, Discussion and Conclusion. Finally, a critique of the article is presented. The journal club coordinator facilitates discussion of strengths and weakness of the study. The issue is then related to the local context and possible solutions discussed. Residency faculty supervises the session and provides feedback.

#### Evaluation

The presenting resident is evaluated by the faculty and senior residents on a standard evaluation form containing ten areas including the degree to which problem background relevant to Pakistan was provided, whether the resident was able to apply the article's information to a relevant context, and whether responses to queries were answered satisfactorily. Evaluations are compiled quarterly and are included in the overall evaluation of the resident.

## Methods

The primary outcome measures for this study were:

1. To check success of CMR-JC through 2 success criteria reported in the literature (i.e. continuous existence for 2 years or longer and an estimated attendance of 50% or higher)

2. To check through a survey whether the alumni and residents are satisfied with CMR-JC activity.

Hence the study included two stages; the first during which a review of the format and characteristic of CMR-JC encompassing the period from September15, 1999 to September 15, 2005 was carried out. This was done to match for the above mentioned criteria for effective journal clubs coming from the literature search. The Second stage comprised a cross-sectional survey for assessment of participant satisfaction with the CMR-JC (see Additional file 1 for questionnaire used in the survey).

### 1. Assessment of CMR-JC characteristics

Journal club presentations were identified through the departmental record of journal club articles. Collected records included full text articles, abstracts for work-in-progress (residents' research projects are presented in CMR-JC for feedback and refinement and for these work-in-progress sessions a one or two pager document, similar to an abstract is circulated to the department at least 2 days prior to CMR-JC) and placement presentations. The data collected were verified by the alumni, faculty and residents of the CMR Program for accuracy and completeness. We reviewed the characteristics of the CMR-JC with regard to presence or absence of the earlier mentioned criteria and associated factors of successful journal clubs as well as annual number (i.e. how many journal clubs were held during the study period), type (whether it was an article presentation from a journal, sharing of placement experiences or a research protocol, or work-in-progress session), name of the journal in which the article was published and topic area of presentations (e.g. communicable diseases, non communicable diseases, reproductive health, nutrition, health systems). All of these presentations and articles were in English.

### 2. Assessment of CMR-JC by the residents

An anonymous questionnaire was mailed to the alumni working outside the department and circulated directly to current residents. Inclusion criteria encompass all current residents; all alumni; all who did not complete residency but who have attended CMR-JC for at least one year and have presented at least thrice in the forum. Since the start of CMR-JC, a total of 32 residents have gone through the residency program and we were able to contact more than 90% of them. Of the 29 resident/alumni contacted, 27 responded to the structured questionnaire.

The questionnaire contained 23 questions. (Table 3) Responses to 13 questions were recorded on a 5-item Likert scale (1 = strongly disagree, 5 = strongly agree). Responses to the remaining 10 questions were to be chosen from 4 given options as well as a 5^th ^"others" option. These questions were pertaining for example to the preferred method of continuing education, important reasons for attending CMR-JC and opinion regarding mock (practice) presentations and journal club coordination. Data entry and analysis was carried out using Microsoft Excel (version 2003) using standard statistical analysis.

## Results

The results of the study are presented in the order of study methodology. First, findings coming through the review of CMR-JC data base and its comparison with international criteria are described, followed by the results of the survey questionnaire.

### a) Findings of CMR-JC database review

#### General characteristics

Table 1 describes the characteristics of CMR-JC during the period September 15, 1999 to September 15, 2005. During the study period the journal club was conducted for a total of 218 hours. Maximum number of presentations (45) was made during 2004. Total number of journal articles presented was 170(77.9%) followed by work-in-progress sessions 25(11.4%). Resident's research protocols accounted for 7(3.2%) of presentations given. Topics relating to communicable diseases were presented 47 (21.5%) times followed by health systems 32(14.6%) and reproductive health & demography related topics 24(11.0%). Articles from WHO Bulletin, British Medical Journal, American Journal of Public Health, Health Policy and Planning, Lancet and Journal Pakistan Medical Association were the most commonly presented in the journal club. Most of these were accessible to residents on the internet.

#### Effectiveness attributes and associated factors

Attributes of successful journal clubs and their associated factors (documented in literature from various medical disciplines) are mentioned in table 2 along with their presence or absence in CMR-JC. The table illustrates that CMR-JC has run continuously for about six years. Both criteria of successful journal clubs were present in CMR-JC. Regarding the associated factors of success, except for use of structured article review checklist, all the factors were present in CMR-JC. Faculty participation and support in the CMR-JC was regular in case of residency program's faculty but was irregular for other faculty of the department.

### b) Findings of participant survey

#### Characteristics of respondents

Four residents each were from first and fourth years and 2 residents were in second year. At the time of survey there was no third year resident in the program. Fifteen of the respondents were alumni of the residency program. Interestingly, more than 10% of the respondents were those who have not completed their residency but have responded to the survey questionnaire. Twelve (42.3%) of the respondents were female.

#### Preferred method of continuing education and reasons for attending CMR-JC

The participants rated journal club (33.3%) as the second most preferred method of continuing education besides courses (37.0%) offered by the department. Learning of critical appraisal skills (44.4%), keeping up with current literature (29.6%), and requirement of mandatory attendance (21.4%) were cited as the most important reasons for attending CMR-JC.

#### Knowledge, skills and behaviour assessment

**Figure 1:**
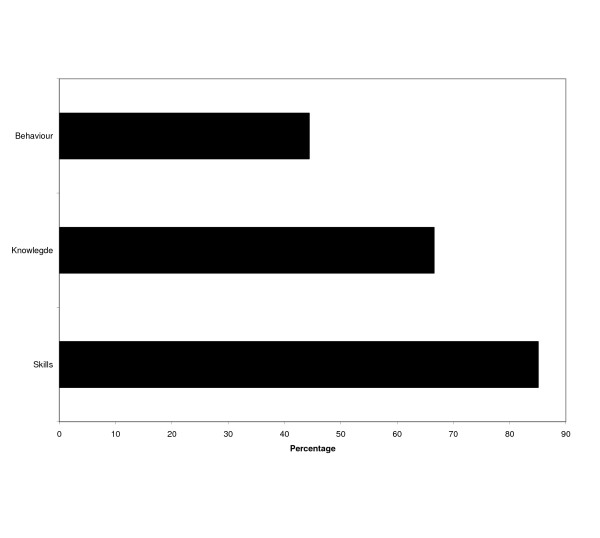
Self-assessed improvement in knowledge, skills and behaviour resulting from participation in CMR-JC conducted at a private university in Karachi Pakistan from 15 Sept 1999–15 Sept 2005.

The questionnaire asked the respondents to assess themselves in the areas of knowledge (keeping up with current literature), skills (confidence in the ability to critically evaluate the paper) and behaviour (enhancement in reading habits as a result of participating in CMR-JC) as described in Figure 1. More than 85% of the respondents perceived a change in critical appraisal skill as a result of attending CMR-JC, followed by an increase in knowledge (66.6%). Less than half (44.4%) the respondents thought that participation in CMR-JC was associated with improved reading behaviour.

#### Effectiveness of the CMR-JC

Table 3 illustrates responses of survey participants on the value of the journal club with regard to the education/learning experience. Majority (89%) of the participants have agreed with the educational value of attending and preparing for CMR-JC. In terms of provision of adequate literature review and stimulus for further reading the agreement level was (88.8%) and (70.3%) respectively. An overwhelming majority (92.5%) agreed with the idea of including a structured article review checklist in the current format.

## Discussion

### Meeting international and local standards

We examined effectiveness criteria of successful journal clubs from various residency programs across the world and compared these with CMR-JC. Our study identified that both of the effectiveness criteria were present in CMR-JC.

We also matched CMR-JC with the factors associated with effectiveness. All but one associated factors of effective journal clubs were found in CMR-JC and this was the use of standard check list for article review. In addition, faculty participation was one attribute which was found partially in CMR-JC (Table 2).

Studies have found that journal clubs benefit from an explicit expectation that residents attend journal club and if journal clubs are highly valued by the program director [4,37,38]. Research has also identified that adult learning experiences are limited by the didactic and lecture modes of teaching. Ebbert and colleagues [1] give examples of how principles of adult learning increase effectiveness of medical education in journal club settings: application of the learning task to the contextual problems, active learner participation, and provision of timely and constructive feedback. The CMR-JC demonstrates all of these characteristics.

Provision of food is associated with long-term, continuous existence of journal clubs [4]. Many clinical disciplines providing food rely on funding from pharmaceutical companies which offer the funds or food as a part of product promotion, or some times it is provided through funding by department. In CMR-JC, food is provided but in a manner that demonstrates resident support of the journal club: the presenting resident uses personal funds to host a breakfast for participants following the session.

### Faculty participation

Regular attendance by faculty correlates with high levels of satisfaction because respected faculty involvement in the teaching process may increase resident participation [32,37,38]. Feedback from the faculty and alumni generates a good debate leading the resident to look into the deeper issues. For the sake of this study, the attribute of faculty participation needed to be viewed in two ways. CMR-JC is successful because the *residency program faculty *participation is always there yet one of the associated attributes of successful journal clubs which is found partially in CMR-JC is faculty participation i.e. the faculty members of the department who are *not *part of Community Medicine Residency Program attend CMR-JC sporadically. This may account for why the study found participants dissatisfied that fewer departmental faculty outside the residency program attend the CMR-JC (table 3).

### Standard checklist for article review

In our survey one of the strongest responses from participants' was in the support for a standard checklist for article review. This was the only success associated factor not found in CMR-JC. These structured article review check lists ask participants to answer questions related to hypothesis, methodology, and application of appropriate statistical tests, results and conclusion for the article. Use of checklist is associated with increased resident satisfaction which can also enhance the value of a journal club [39]. Currently, in CMR-JC the *presenting *resident's understanding of the article is assessed on a structured evaluation form by faculty and senior residents. These evaluations are added to the presenter's formative evaluation. We suggest that the article review checklists can serve to assess all *attending *residents' understanding of the presented article, forming a part of their formative evaluation as well.

### Self-assessment of knowledge, skills, behaviours

The study evaluates the importance of journal clubs from the residents' perspective. Residents valued the experience and perceived that specific gains resulted from their participation in the club. Over 85% of respondents perceived a change in critical appraisal skills and over 65% indicated an increase in knowledge in the topic areas. In addition, residents indicated that they value attending and preparing for CMR-JC. They were satisfied with the timing of CMR-JC and agreed that presentation of their research work helps them to refine it. Self-assessment may not fulfil the criteria of excellent evidence as do some of the analytical study designs such as before and after, cohort and intervention studies including randomized controlled trial. Since results of some of these analytical and intervention studies do suggest improvement in knowledge, skills and behavior through courses based on the critical appraisal approach, [3,7,14,19,40,41] assessment by more methodologically rigorous study designs would be more effective in demonstrating the perceived increases in CMR-JC.

Another limitation might be the design of the Likert scale 'neutral' response. For some questions as many as 29% indicated neutrality which hampers a clear measure of participant opinion. We included the 'neutral' option in the scale because of the sensitivity of some questions (for example, those pertaining to faculty participation.).

## Conclusion

Good public health decisions depend on combining good information (routinely collected statistics) with good research evidence [42]. Further, the core competencies required by the College of Physicians and Surgeons in Pakistan (Pakistan's fellowship training accreditation body) include the ability to interpret data, the ability to understand the implications of research findings and familiarity with recent knowledge in the field. The findings of this study support that journal clubs can be effective in the training of Community Medicine (Public Health) residents to meet these core competencies.

This study shows that a journal club can help residents develop the habit of staying updated on the significant literature that is published every year in medical science. Against the backdrop of a general resource constraint in developing countries, CMR-JC has made effective use of information technology for selecting articles from reputed international journals online. Given the free access to 17,000 scientific journals online, by Higher Education Commission (HEC) of Government of Pakistan; public sector medical universities can utilize the approach of journal club to train postgraduate trainees to make their practice evidence based [43]. This exemplary effort on the part of the HEC could provide a model for governments in countries where there are similar resource constraints.

Having shared the experience of a journal club which has thrived for more than six years and is popular among its participants, we would like to recommend that CMR-JC has shown a format for organizing and successfully running journal clubs in a developing country's setting with resource constraints and limited access to print publications. This design can be used by any residency program in similar settings for teaching and assessing resident's ability to critically appraise research as a core competency. Moreover this format can also be used for assessing the overall efficacy of journal clubs in meeting their objectives.

### Further research

Currently in Pakistan the existence and effectiveness of journal club meetings in postgraduate medical education is unreported. We achieved greater than 90 percent response rate, yet the sample represents only one medical university from Pakistan. Therefore, generalization requires caution. We recommend the study be replicated in other public health residency programmes in Pakistan and internationally. The literature also suggests that regular assessment surveys of journal clubs are recommended and we support this recommendation.

A majority of participants in this study have recommended improved faculty participation in the journal club. It would be interesting and helpful to further explore what residents would like to see regarding faculty participation. It would also be valuable to look at faculty perspective regarding participation in CMR-JC.

While this study has particular relevance for Pakistan, continued assessment of journal clubs, enhanced by the learning from this study, can contribute to public health-related journal club establishment and assessment wherever public health practitioners are trained.

## Competing interests

The author(s) declare that they have no competing interests.

## Authors' contributions

SA participated in the design of the study, collected and analyzed data and drafted the manuscript. MMK conceived the idea, supervised the study and gave intellectual input in the manuscript. All authors read and approved the final manuscript.

## Pre-publication history

The pre-publication history for this paper can be accessed here:



## Supplementary Material

Additional file 1 Survey Questionnaire. Survey questionnaire sent to the residents and alumni of Community Medicine Residency Program to meet the second objective of the study. Click here for file

## Figures and Tables

**Table 1:** General characteristics of CMR-JC conducted at a private university in Karachi Pakistan from 15 Sept 1999 –15 Sept 2005

**Number of presentations annually**	**n(%)**
1999 (Sept–Dec only)	14 (6.4)
2000	34 (15.5)
2001	30 (13.7)
2002	33 (15.1)
2003	35 (16.0)
2004	45 (20.6)
2005 (till 15 Sept)	29 (13.3)
**Type of work presented**	
Articles	170 (77.9)
Work-in-progress sessions	25 (11.4)
Literature synthesis /protocol	7 (3.2)
Placement presentations	5 (2.2)
Training experiences	4 (1.8)
Alumni presentations	4 (1.8)
Faculty presentations	4 (1.8)
**Number of presentations by topic area**	
Communicable diseases	47 (21.5)
Non-communicable diseases	19 (8.7)
Health Systems/Management issues	32 (14.6)
Reproductive Health/Demography	24 (11.0)
Mental Health	16 (7.3)
Environmental Occupational Health	15 (6.8)
Injuries	10 (4.5)
Nutrition	6 (2.7)
Miscellaneous	50 (22.9)

N = Total number of presentations = 218

**Table 2:** Effectiveness attributes and associated factors of journal clubs and their presence in CMR-JC conducted at a private university in Karachi Pakistan from 15 Sept 1999 –15 Sept 2005

**Effectiveness attribute**	**Presence in CMR-JC**
>2 years of existence without periodic abandonment	+
>50% attendance of the expected audience	+
**Associated factors of effectiveness**	
Explicit written learning objectives	+
Mandatory attendance	+
Designated club leader	+
Formal teaching of critical appraisal skills	+
Independence from faculty journal club	+
Regular attendance by faculty*	±
Support by program director	+
Smaller residency program	+
Use of adult learning principles	+
Provision of food	+
Use of a structured checklist for article review	-

*The residency program faculty attendance was regular but other departmental faculty attendance was not regular

**Table 3:** Effectiveness of CMR-JC conducted at a private university in Karachi Pakistan from15 Sept 1999–15 Sept 2005 in the opinion of residents and alumni

**Variable**	**Disagreement**	**Neutral**	**Agreement**
Educational value of attending CMR-JC	3(11.1)	0(0.0)	24(88.8)
Educational value of preparing for CMR-JC	1(3.7)	2(7.4)	24(88.8)
CMR-JC provide good review of literature	0(0.0)	3(11.1)	24(88.8)
CMR-JC provide stimulus to further reading	2(7.4)	6(22.2)	19(70.3)
Presentation at CMR-JC refine research	4(14.8)	6(22.2)	17(62.9)
Satisfaction with residency program faculty participation	7(25.9)	8(29.6)	12(44.4)
Satisfaction with departmental faculty participation	22(81.4)	3(11.1)	2(7.4)
Timing of CMR-JC is appropriate	3(11.1)	3(11.1)	21(77.7)
Overall satisfaction with CMR-JC format	5(18.5)	8(29.6)	14(51.8)
Introduction of a standard check list for article review would be helpful	0(0.0)	2(7.4)	25(92.5)

N = 27
