# Supplementary material for: Do community medicine residency trainees learn through journal club? An experience from a developing country
Source: BMC Med Educ. 2006 Aug 22;6:43. doi: 10.1186/1472-6920-6-43 (PMC1564014; doi:10.1186/1472-6920-6-43)
Supplement: Additional file 1 — Survey Questionnaire. Survey questionnaire sent to the residents and alumni of Community Medicine Residency Program to meet the second objective of the study. [file 1472-6920-6-43-S1.doc]

**Survey Questionnaire**

1. Date __________
2. Status: a) Resident Year of residency _________

b) Alumnus Year of passing out _________

| ***S.No*** | ***Question*** | ***Coding Categories*** | ***Answer*** |
| --- | --- | --- | --- |
| Q1 | In your opinion what is the most important goal of a journal club? | 1. to keep up with current  literature  2. to teach critical reading skills  3. to impact clinical practice  4. to improve reading habits  5. any other (specify) |  |
| Q2 | Which of the above goal/goals is achieved by CMR JC? | 1. to keep up with current  literature  2. to teach critical reading skills  3. to impact clinical practice  4. to improve reading habits  5. any other (specify) |  |
| Q3 | You found attending CMR JC to be of educational value | 1. strongly disagree 2. disagree 3. neither agree nor disagree 4. agree 5. strongly agree |  |
| Q4 | You found preparing for CMR JC to be of educational value | 1. strongly disagree 2. disagree 3. neither agree nor disagree 4. agree 5. strongly agree |  |
| Q5 | In your opinion CMR JC helped in development of research protocols | 1. strongly disagree 2. disagree 3. neither agree nor disagree 4. agree 5. strongly agree |  |
| Q6 | Has your research work /dissertation come out of a journal club presentation? | 1. yes  2. no |  |
| Q7 | Presentation at CMR JC helped refine your research work? | 1. strongly disagree 2. disagree 3. neither agree nor disagree 4. agree 5. strongly agree |  |
| ***S.No*** | ***Question*** | ***Coding Categories*** | ***Answer*** |
| Q8 | In your opinion CMR JC provides good review of public health related literature? | 1. strongly disagree 2. disagree 3. neither agree nor disagree 4. agree 5. strongly agree |  |
| Q9 | In your opinion CMR JC provides stimulus to further review a topic | 1. strongly disagree 2. disagree 3. neither agree nor disagree 4. agree 5. strongly agree |  |
| Q10 | In your opinion CMR JC facilitate development of critical appraisal skills | 1. strongly disagree 2. disagree 3. neither agree nor disagree 4. agree 5. strongly agree |  |
| Q11 | Please indicate the most important reason/reasons for your decision to attend CMR JC | 1.mandatory attendance  2. social gathering  3.to keep up with current  literature  4. to learn critical reading skills  5. to impact clinical practice  6. to learn  epidemiology/biostatistics  7. to improve presentation skills  Others *(specify)_____________* |  |
| Q12 | Which key journals would you like to be made mandatory for the residents to review? | Name of journal  1.  2.  3.  4.  5. |  |
| Q13 | Which of the methods for continuing education do you prefer the most? | 1. courses  2. journal club  3. workshops  4. conferences  Others (specify)__________ |  |
| Q14 | Do you think that introduction of a standard check list for review of different segments of an article would be helpful to improve resident participation? | 1. strongly disagree 2. disagree 3. neither agree nor disagree 4. agree 5. strongly agree |  |
| ***S.No*** | ***Question*** | ***Coding Categories*** | ***Answer*** |
| Q15 | In your opinion the current format* of CMR JC is satisfactory? | 1. strongly disagree 2. disagree 3. neither agree nor disagree 4. agree 5. strongly agree |  |
| Q16 | In your opinion is residency program faculty participation in CMR JC satisfactory? | 1. strongly disagree 2. disagree 3. neither agree nor disagree 4. agree 5. strongly agree |  |
| Q17 | In your opinion is CHS (departmental) faculty participation in CMR JC satisfactory? | 1. strongly disagree 2. disagree 3. neither agree nor disagree 4. agree 5. strongly agree |  |
| Q18 | Have you ever presented a mock presentation before JC? | 1. yes  2. no |  |
| Q19 | In your opinion is presenting a mock presentation a valuable exercise in preparing for CMR JC? | 1. strongly disagree 2. disagree 3. neither agree nor disagree 4. agree 5. strongly agree   99. Not applicable |  |
| Q20 | Have you ever been assigned the responsibility of CMR JC coordination? | 1. yes  2. no |  |
| Q21 | In your opinion is/was coordinating JC was a helpful experience to your own residency training? | 1. strongly disagree 2. disagree 3. neither agree nor disagree 4. agree 5. strongly agree   99. Not applicable |  |
| Q22 | Is the timing of journal club (i.e. the first thing in the morning) appropriate? | 1. strongly disagree 2. disagree 3. neither agree nor disagree 4. agree 5. strongly agree |  |
| Q23 | Recommendations to improve over all quality of CMR JC |  | |

* Format includes once a week occurrence from 8-9 am, presentations on rotations, regular feedback by residency director and coordinator, some times attended by other faculty members, evaluation of presentation by director, coordinator and senior residents on a prescribed form. Besides article presentations, work in progress sessions, sharing of training experiences and alumni presentations are also included in CMR JC.
